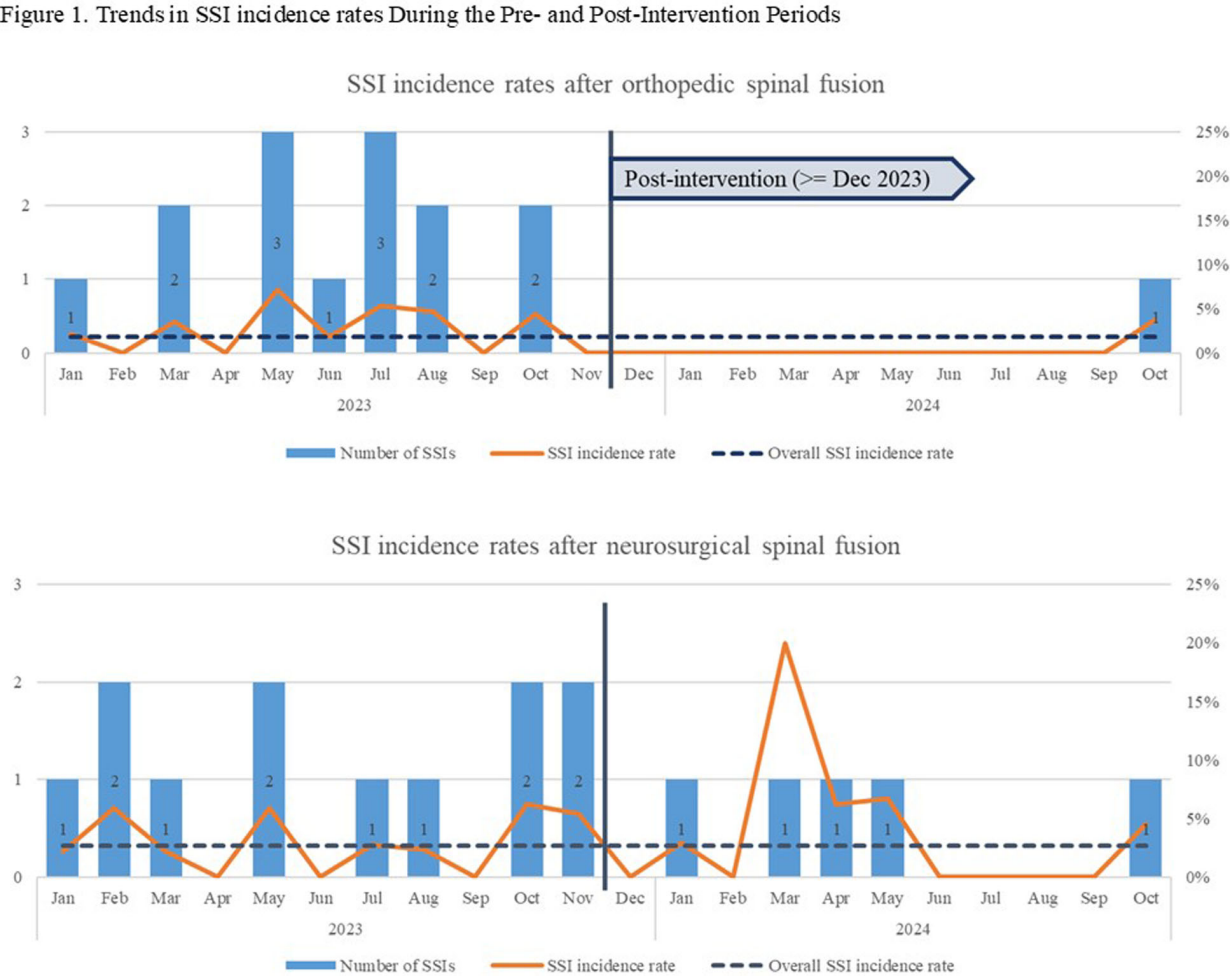# 51 Carbapenem-resistant organism and Candida auris cases among non-nursing home congregate facility residents in New York City, 2019–2024

**DOI:** 10.1017/ash.2026.10712

**Published:** 2026-06-23

**Authors:** Subeen Moon, Mijung Kim, Hyunju Lee, Miseo Kim, Soyeon Park, Jeeyoon Kim, Jiwon Jung, Yong Pil Chong

**Affiliations:** 1 Office for Infection Control, Asan Medical Center, Seoul, Korea

## Abstract

**Background:** Surgical site infection (SSI) following spinal fusion is a major complication that increases mortality and healthcare costs, with Staphylococcus aureus being the most common pathogen. Implementation of an SSI prevention bundle has been reported to significantly reduce SSI rates. This study evaluated changes in SSI rates among orthopedic spinal fusion patients after applying a prevention bundle that included S. aureus decolonization therapy and compared the results with non-intervention neurosurgical spinal fusion patients during the same period. **Methods:** At a tertiary care hospital in Seoul with 2,432 beds, an enhanced SSI prevention bundle including S. aureus decolonization therapy was implemented for all orthopedic spinal fusion patients beginning in December 2023. Decolonization consisted of a chlorhexidine shower the day before or the day of surgery and intranasal application of povidone-iodine within two hours before surgery. Additional bundle components included the use of colored inner gloves during auto-graft bone preparation to detect glove tearing, and additional sterile drapes when using a C-arm. The intervention was accompanied by staff education, promotion activities, awareness surveys, and ongoing monitoring. SSI rates were compared between the pre-intervention period (January-November 2023) and post-intervention period (December 2023 – October 2024) in orthopedic patients and further compared with neurosurgical surgery to evaluate the intervention’s effectiveness. Pediatric patients who underwent scoliosis surgery and patients with infections present at the time of surgery (PATOS) were excluded from the study. Diagnosis was based on the updated 2023 CDC/NHSN (National Healthcare Safety Network) definitions. **Result:** In the orthopedic department, the SSI rates significantly decreased from 2.65% (14/529) before the intervention to 0.34% (1/295) after the intervention (p=0.01). In the non-intervention neurosurgery department, there was no significant difference in SSI rates between the two periods (3.05% [12/393] vs. 2.16% [5/231], p=0.62). Although the post-intervention SSI rates in orthopedics were lower than that in neurosurgery (0.34% vs. 2.16%), the difference was not statistically significant (p=0.09). The SSI rates caused by Staphylococcus spp. in orthopedics decreased from 2.27% (12/529) before the intervention to 0% (0/295) after the intervention (p=0.01). In neurosurgery, there was no significant change in the incidence of SSIs caused by Staphylococcus spp. between the two periods (1.53% [6/393] vs. 1.30% [3/231], p<0.99). **Conclusion:** The enhanced SSI prevention bundle, which included S. aureus decolonization therapy, effectively reduced the SSI rate in orthopedic fusion surgeries. This effect was not observed in the neurosurgery department without the intervention, supporting the effectiveness of the implemented bundle.

**Figure:**